# Retrospective Analysis of Rectal Endoscopic Submucosal Dissection at Ordensklinikum Linz and Kepler Universitätsklinikum Linz

**DOI:** 10.3390/jcm13123530

**Published:** 2024-06-17

**Authors:** Nikolaj Swiridoff, Alexander Ziachehabi, Friedrich Wewalka, Georg Spaun, Vedat Alibegovic, Rainer Schöfl

**Affiliations:** 1 Interne IV (Gastroenterology), Ordensklinikum Linz Barmherzige Schwestern, 4010 Linz, Austriarainer.schoefl@ordensklinikum.at (R.S.); 2 Clinic for Internal Medicine 2—Gastroenterology and Hepatology, Endocrinology and Metabolism, Nephrology, Rheumatology, Kepler University Hospital, 4020 Linz, Austria; 3 General and Visceral Surgery, Ordensklinikum Linz Barmherzige Schwestern, 4010 Linz, Austria; 4 Pathology, Ordensklinikum Linz Barmherzige Schwestern, 4010 Linz, Austria

**Keywords:** endoscopic submucosal dissection, endoscopic dissection, rectum, early neoplasia, complications, Austria

## Abstract

**Background and study aim**: Endoscopic submucosal dissection is a minimally invasive endoscopic procedure for the removal of neoplastic benign and early malignant lesions in the gastrointestinal tract. In this study, we analyse the success and safety of rectal ESD at Linz hospitals, focusing on a specific endoscopist. Additionally, we examine whether there is a learning curve regarding success parameters. **Methods**: This retrospective study included all 102 patients who underwent endoscopic submucosal dissection of the rectum by a defined endoscopist at Ordensklinikum Hospital and Kepler University Hospital between December 2010 and May 2021. With the collected data, a descriptive statistic was carried out and regression analyses were performed. **Results**: The en bloc resection rate was 78.4% and the rate of lesions removed in healthy tissue was 55.6%. The average procedure time was 179 min and the complication rate was 7.8%. In total, 26.4% of cases showed carcinoma; in 25.9% of these cases, an oncologically curative resection was achieved with ESD. Follow-up data were available for 61.1% of cases, with recurrence being diagnosed in 3.6% of cases. A learning curve was observed regarding the rate of lesions removed in healthy tissue and the procedure time, but not regarding the en bloc resection rate. **Conclusions**: Endoscopic submucosal dissection is a safe method for the removal of large rectal adenomas and early carcinomas. The en bloc resection rate of the analysed procedures is within the range of comparable European studies. The rate of lesions removed in healthy tissue is below the R0 resection rate of the comparative literature; however, a learning curve could be observed in this parameter.

## 1. Introduction

Endoscopic submucosal dissection (ESD) was originally developed in Japan for the treatment of early gastric carcinoma [1]. While it has already become a standard minimally invasive endoscopic procedure in many Asian countries [1], its role in the treatment of colorectal neoplasia is not yet clearly defined in the Western world, and there is still a lack of experience [2].

With ESD, even larger lesions (>20 mm) can be resected in one piece (en bloc) [3]. This means that endoscopic treatment can be considered curative even if carcinoma is detected, provided that R0 resection was successful and the lesion is at low risk (submucosal invasion depth < 1000 µm, no lymphovascular invasion, no venous invasion, good to moderate differentiation, no tumour budding) [3]. This is a significant advantage over endoscopic mucosal resection (EMR), which does not allow for the resection of larger lesions in one piece, i.e., only “piecemeal”.

On the other hand, ESD is associated with an increased risk of complications compared to EMR [4]. In addition, ESD is technically more demanding and time-consuming [5]. The success of ESD depends on the experience of the endoscopist, with the rates for en bloc and R0 resection increasing with the number of procedures performed, while the average operating time decreases [2,6].

The aim of the present study was to analyse the success and safety of ESD in the rectum in hospitals in Linz, Austria. Furthermore, we investigated whether, as in other studies, a learning curve could be observed with regard to success rates, complications, and procedure duration.

## 2. Patients and Methods

### 2.1. Data Collection

Data were collected retrospectively using electronically stored medical reports, endoscopy protocols, endoscopy findings, imaging findings, pathology findings, and tumour board protocols.

### 2.2. Patient Collective

This study included 102 consecutive patients treated with an ESD by the defined endoscopist in the period from 1 December 2010 to 29 February 2020 at one of the two Ordensklinikum hospitals or in the period from 1 March 2020 to 21 May 2021 at Kepler University Hospital. Prior to the first interventions included in this study, the endoscopist had already gained experience by performing ten ESDs on the stomach. Before that, the examiner had spent a month in Japan in 2009 and completed several training sessions on pig stomachs and live pigs.

### 2.3. Parameters

In addition to demographic data, the date and hospital of the intervention and the localization of the tumours (proximal, middle, distal third of the rectum) were recorded as a parameter known prior to the intervention. In addition, we differentiated whether the tumour was a recurrent polyp, or a neuroendocrine tumour (NET) known prior to the intervention.

**Figure 1:**
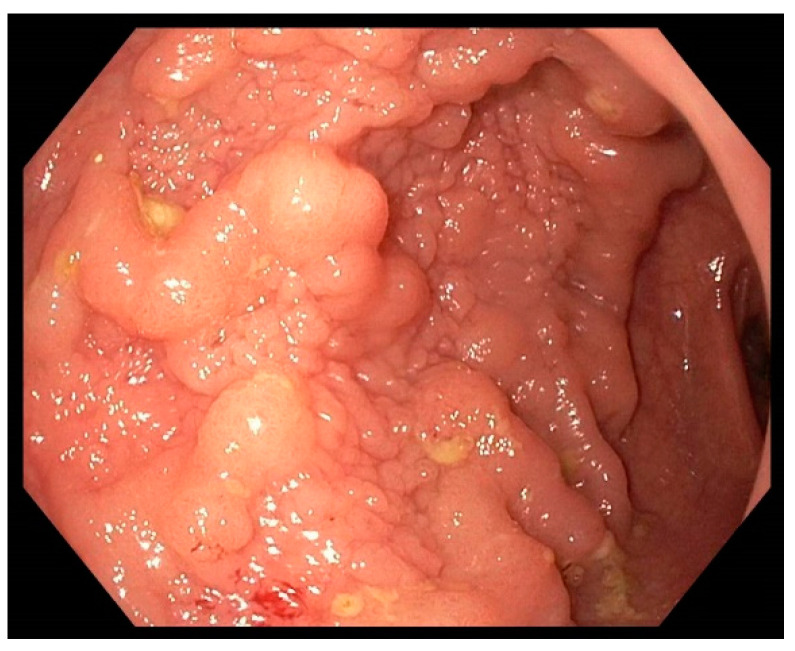
Laterally spreading tumour, granular type (nodular-mixed) in the proximal rectum. The histologic workup showed an HGIN.

**Figure 2:**
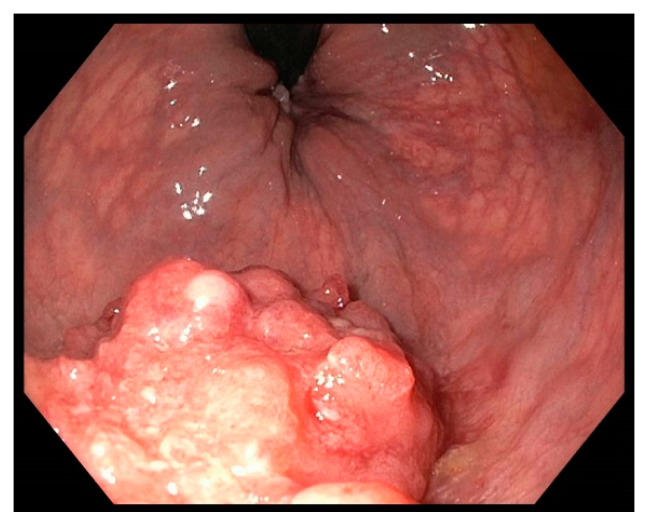
Laterally spreading tumour, non-granular type in the distal rectum. The histologic workup revealed an adeno-ca. pT1 (sm3) G2 L1 V0.

Figure 1 and Figure 2 show examples of endoscopic images of lesions that were evaluated in this study.

The procedure-related parameters recorded were whether the lesion was removed en bloc and in healthy tissue, whether a complication occurred, and the duration of the procedure. The prerequisite for removal in healthy tissue was an en bloc resection. In the case of a carcinoma, only a histologically confirmed R0 resection was considered a removal in healthy tissue. In the case of an adenoma, if the marginal zone could not be assessed histologically, for example due to coagulation artifacts, the lesion was considered to have been removed in healthy tissue if the endoscopy findings indicated removal in the macroscopically healthy tissue.

For complications, a distinction was made between bleeding, perforations, and other complications. In addition, a classification was made according to minor and major complications. A complication was considered a major complication if a hospital readmission after discharge, a re-endoscopy with haemostasis or clipping of a perforation, an operation, the administration of blood transfusion or catheter embolization was necessary.

The time from the endoscopy protocol was used as the duration of the procedure, meaning the time the patient spent in the endoscopy room.

**Figure 3:**
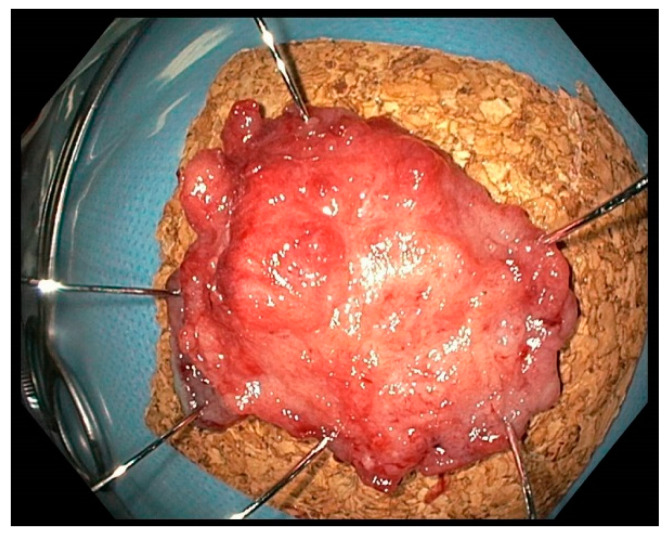
ESD specimen of the polyp from Figure 2 mounted on cork. Histological examination revealed an adeno-ca. pT1 (sm3) G2 L1 V0 R1.

The histological findings were used to determine the type and size of the lesion and, in the case of carcinoma, the depth of submucosal invasion, grading, tumour budding, lymph vessel invasion, venous invasion, and perineural sheath invasion. As the size of the lesion, the specimen length measured by pathology was used. In cases where this was missing, for example, when the specimen was present in multiple pieces due to an unsuccessful en bloc resection, the size estimation of the endoscopist was used. Figure 3 shows an ESD specimen of a suspicious polyp removed in this study, mounted on cork.

In addition, it was determined whether the ESD was oncologically curative and whether a subsequent operation for further tumour removal was recommended and performed.

The occurrence of recurrence was evaluated on the basis of the first follow-up examination at least 90 days after the ESD and subsequent reports in patients without subsequent surgery.

### 2.4. Statistics

In order to identify a possible learning curve in the results, the 102 patients were divided chronologically into four cohorts. The first three cohorts included 27 patients each, and the last one included 21 patients.

All data of the continuous variables were checked for normal distribution (test of normality: Kolmogorov–Smirnov with Lilliefors significance correction, type I error = 10%) and in the case of normal distribution, also for variance heteroscedasticity (Levene test, type I error = 5%). Since in no case could both normal distribution and variance homogeneity be determined, all cohort comparisons of continuous variables, as well as of variables measured on ordinal scales, were performed by a non-parametric analysis of variance (Kruskal–Wallis test, followed by Nemenyi’s multiple comparisons). Data of categorical variables were compared by the chi-square test (exact or with Monte Carlo simulation, with the provision of adjusted residuals).

The influence of the endoscopist’s experience with ESD in general, and the endoscopist’s experience with ESD on the rectum in particular, on en bloc resection and removal in healthy tissue was investigated using logistic regression analyses in a multivariate model.

Since the type I error was not adjusted for multiple testing, the results of inferential statistics are descriptive only, and the use of the term “significant” in the description of the study results always reflects only a local *p* < 0.05 but no error probability below 5%. Statistical analyses were performed using the open-source R statistical software package, version 4.0.5 (The R Foundation for Statistical Computing, Vienna, Austria). The influence of a recurrent lesion on the rate of en bloc resections and lesions removed in healthy tissue was investigated using the chi-square test. These calculations were performed using Microsoft Excel 365.

## 3. Results

### 3.1. Patient Collective

In total, 102 patients were treated with an ESD by an endoscopist during the data collection period. Sixty (58.8%) of these patients were male and 42 (41.2%) female. The mean age of the patients was 66.3 years. 

Thirteen lesions (12.9%) were located in the proximal rectum, 31 (30.7%) in the mid-rectum, and 57 (56.4%) in the distal rectum. In one case, no information could be obtained from the patient records. 

In 17 cases (17.0%), it was a recurrent polyp. In 2 cases, ESD was performed as a post-resection, whereby in both cases there was no residual histological tumour in the post-resected tissue.

### 3.2. Rate of En Bloc Resections

**Figure 4:**
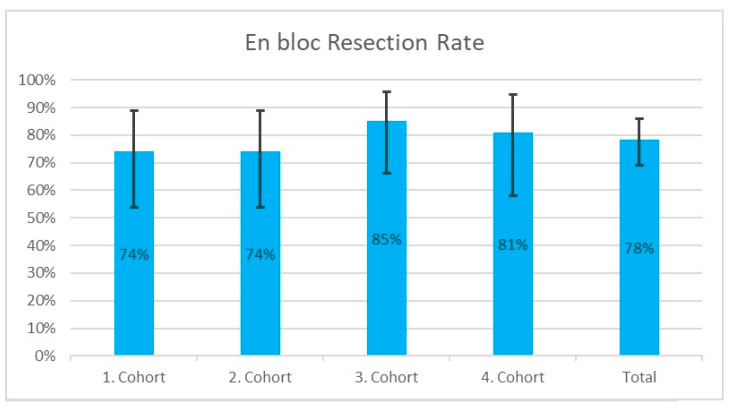
En bloc resection rates in the individual cohorts and in the overall collective. The respective 95% confidence intervals are plotted vertically around the en bloc resection rates.

En bloc resection was achieved in 78.4% (80/102) of cases. The rates of en bloc resection in the individual cohorts are plotted in Figure 4 together with their respective 95% confidence intervals.

If a recurrent polyp existed, we recorded a statistically non-significant decrease in the en bloc resection rate to 70.6% (12/17) compared to 79.5% (66/83) in cases without a recurrent polyp.

Neither the cohort comparison nor the regression analysis showed a statistically significant learning curve.

### 3.3. Rate of Lesions Removed in Healthy Tissue

**Figure 5:**
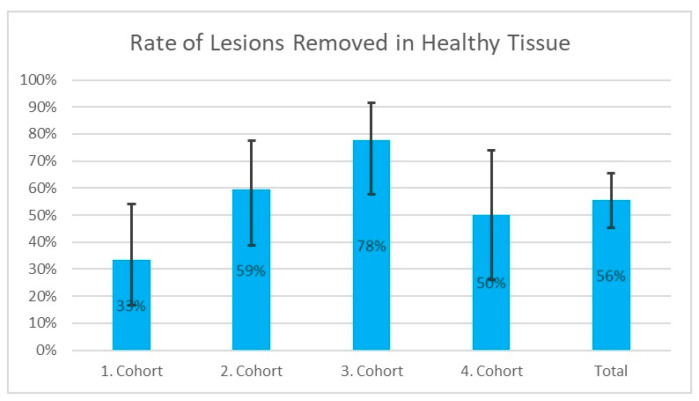
Proportion of lesions removed in healthy tissue in the individual cohorts and in the overall collective. The 95% confidence intervals are plotted vertically around the respective values.

Removal in healthy tissue was achieved in 55.6% (55/99) of cases. Three cases were not analysed in this regard: two cases involved post-resection without a tumour remnant, and one case showed a hyperplastic polyp on histological examination. Figure 5 shows the proportion of lesions removed in healthy tissue in the various cohorts and in the overall collective.

As with the en bloc resection rate, there is a statistically non-significant trend towards a lower rate of recurrent polyps removed in the healthy tissue of 47.1% (8/17) vs. 57.3% (47/82) for procedures without recurrent polyps.

In contrast to the en bloc resection rate, we were able to demonstrate a statistically significant learning curve in the learning curve analysis in the cohort comparison (*p* = 0.010) and also in the logistic regression analysis (*p* = 0.033). In the regression analysis, the number of ESDs previously performed on the entire gastrointestinal tract by this endoscopist had a statistically more significant influence on the removal of a lesion in healthy tissue (*p* = 0.033) than the number of ESDs previously performed, specifically on the rectum (*p* = 0.053).

### 3.4. Intervention Time

**Figure 6:**
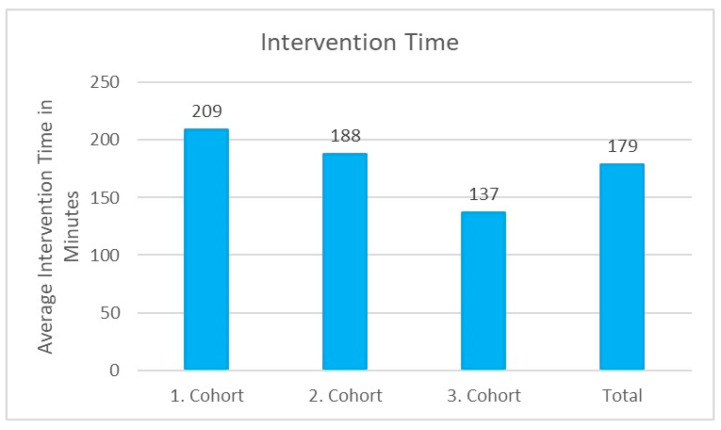
Intervention times in the different cohorts and in the overall collective.

The intervention time was recorded in 79 cases; data for cohort 4 are completely missing. The average intervention time was 179 min. Figure 6 shows the average intervention times in the different cohorts and in the overall collective.

In the statistical analysis, the cohort had a significant influence on the duration of the interventions (*p* = 0.033).

### 3.5. Complications

A complication occurred in 7.8% (8/102) of cases. The complications were four post-interventional bleedings, three perforations, and one post-polypectomy syndrome. According to their severity, four complications (50%) were classified as minor and four (50%) as major complications. Occurrence (*p* = 0.855), type (*p* = 0.529), and severity (*p* = 0.314) did not depend on the cohort in the statistical analysis. All complications were successfully treated conservatively or by re-endoscopy. One patient died of a bilateral pulmonary embolism with right heart decompensation 7 days after the procedure, which was temporally related to the ESD but not casually.

### 3.6. Histological Characterisation

The size of the lesions in relation to the cohort is presented in Table 1. The average size of the lesions was 45 mm (σ = 25.95 mm) (all lesions evaluated) and ranged from 10 mm to a maximum of 152 mm. There was no statistically significant dependence on the cohort (*p* = 0.111). 

Table 2 shows the type of lesion found in the specimen. Excluding the NET already known pre-interventionally and the two post-resections, carcinoma was present in 29% (27/93) of cases.

### 3.7. Further Treatment

Neuroendocrine tumours could be resected oncologically curatively in 85.7% (6/7) of the cases; for T1-adenocarcinomas, this was successful in 29.1% (7/24) of cases. If the ESD was not oncologically curative, surgery was recommended in 73.7% (14/19) of cases. Information regarding this matter could not be obtained for one patient. In five cases, patients were advised against surgery after weighing up the risks of lymph node metastases, morbidity and mortality caused by the surgical procedure and any comorbidities, as well as tumour-independent life expectancy. In the 14 cases in which subsequent surgery was advised, surgical resection following ESD was performed in 11 cases (78.6%). In the remaining three cases, the patients refused.

### 3.8. Follow-Up

Of the 90 patients who did not undergo surgical resection following ESD and who did not die shortly after the ESD, a follow-up examination at least 90 days after ESD was evaluated in 55 cases (61.1%). In 3.6% of these cases (2/55), a recurrence or residual was diagnosed. These were both adenomas, both of which could be removed during the follow-up examination.

In five cases, a recurrence was detected after the first check-up, for example, through another follow-up examination performed at the same hospital or due to a cancer diagnosis treated at the same hospital. Three of these were LGINs, including the case in which an LGIN had already been removed during the first follow-up. In another case, lung metastases occurred later. This was an ESD of an adenocarcinoma that had been assessed as curative. In another case, liver and skin metastases developed. This was a non-curative ESD of an adenocarcinoma in which surgical resection was not performed due to comorbid multiple myeloma.

## 4. Discussion of the Learning Curve

When interpreting the learning curves, it must be considered that the difficulty of the cases increases over time. Due to this allocation effect, it is possible that a learning curve exists but is not statistically detectable.

In our data, this may have been the case regarding the rate of en bloc resections. However, it must also be mentioned here that a possible difference in the patient cohort cannot be proven by statistically significant differences in the pre-intervention parameters or the lesion size. It must also be considered that the endoscopist had already gained a certain amount of experience with the method using ESD in the stomach, observation in Japan and training on pig stomachs before the first rectal ESD.

What is striking about our data is that, despite evidence of a general learning curve, the rate of lesions removed in healthy tissue decreased again with the fourth cohort and only performed similarly to the overall collective. The en bloc resection rate also decreased in the fourth cohort compared to the third cohort. The fourth cohort was treated at Kepler University Hospital. Other framework conditions in comparison with the Ordensklinikum hospitals, such as differences in nursing assistance and different selection criteria with more demanding cases, are conceivable explanations, as is a coincidental occurrence of this anomaly. However, the examiner denied a difference in the quality of nursing assistance. On the other hand, the endoscopist subjectively perceived an increase in the difficulty of the cases over the course of the study, but this could not be substantiated by significant differences in the pre-interventional parameters recorded.

In principle, a change in the technical equipment is also a possible explanation for the occurrence or absence of a learning curve. The DualKnife 1.5 mm (Olympus, Tokyo, Japan) was used for ESD. From the beginning of 2019—at the start of the third cohort—the DualKnife J (Olympus) also offered the option of injecting via a knife. This significantly reduced the need to change instruments to a needle during procedures. The introduction of the DualKnife J would, therefore, be expected to have an impact on the procedure time. A flattening of the learning curve regarding the lesions removed in healthy tissue in the fourth cohort cannot be explained by the timing of the change.

## 5. Literature Comparison

There are several comparable European studies [1,4,5,6,7,8] on the success of ESD, in which the endoscopists, as in our study, had little experience with the method at the start of the study. These show en bloc resection rates of 64% to 90%, R0 resection rates of 53% to 80% and complication rates of 7.5% to 11.5%, and in one study, even a perforation and bleeding rate of 18% and 13%, respectively [4]. A prospective multicentre study analysed the current success rates of ESD in Germany [9]. In 380 ESDs on the rectum, an en bloc resection was successful in 90.5% and an R0 resection in 77.1% of cases. The complication rate was 4.5%. Studies from Asian countries have been showing high success rates (88.0%–98.6% en bloc resections) and low complication rates of ESD in the treatment of colorectal lesions for a decade [10,11].

The en bloc resection rate of our study is, therefore, within the range of other European studies in which the endoscopists gained experience with the method during the ongoing study. The rate of R0 resections in the comparative studies, on the other hand, is predominantly higher than our result. However, comparisons are of limited validity because definitions of success and complications vary. The current en bloc and R0 resection rates in Germany [9] are higher than those of our study, but some of them come from centres where the method was already established. It must also be considered that the proportion of carcinomas in our study was relatively high at 26.4% (comparative literature: 0.0–18.4% [1,4,5,6,8,9], one retrospective study excluded malignant lesions [1]). Additionally, in our study, the proportion of recurrence polyps was 17%, while in the comparative literature, it was 5.6% to 8.2% [1,7], excluded [5] or not named [4,6,8,9]. Recurrence polyps are associated with lower en bloc resection rates [12,13]. Therefore, the procedures we analysed possibly had a higher degree of difficulty. Whether ESD was oncologically curative in the case of carcinoma was only reported in one study that included at least five carcinomas. In this study, the rate of oncologically curative ESD was 7.3% [6], significantly lower than the 25.9% achieved in our study. The reported complication rates in the comparative literature are within the range of our results.

The recurrence rate in the comparative literature is 1.8% to 12% [1,3,4,7,8,9]. Our recurrence rate is, therefore, in the lower range of the reported results. This indicates that the relatively low rate of lesions removed in healthy tissue observed by us is of little clinical significance. 

In the comparative literature, a learning curve regarding en bloc resection [2,4,6,8,14], R0 resection [6,14], complications [4,14] and procedure time [2,6] could be demonstrated in many, but not all studies [15]. We were able to demonstrate such a learning curve with regard to procedure time and the rate of lesions removed in healthy tissue, but not for en bloc resections and the occurrence of complications. The latter may be a case number problem.

Overall, our study confirms that ESD is a safe method for the removal of large rectal adenomas and early carcinomas. The selection of patients for whom ESD, instead of EMR, promises a clinical advantage remains a challenge. In addition to patients with large adenomas, who benefit from the lower recurrence rate of ESD in these lesions, there are particularly patients with endoscopically potentially curable early carcinomas, who are spared a surgical procedure by ESD.

## Figures and Tables

**Table 1:** Lesion size in relation to the cohort.

Cohort	1	2	3	4	Total
Average Lesion Size	33 mm	51 mm	48 mm	48 mm	45 mm

**Table 2:** Type of lesions removed. As expected, most of the lesions were LGIN, HGIN and T1 carcinomas.

Type of Lesion	Number	Proportion in %
Hyperplastic polyp	1	1.0
Low-grade intraepithelial neoplasia (LGIN)	30	29.4
High-grade intraepithelial neoplasia (HGIN)	35	34.3
T1 adenocarcinoma	24	23.5
T2/T3 adenocarcinoma	3	2.9
NET	7	6.9
Post-resection without residual tumour	2	2.0
Total	102	100.0

## Data Availability

Anonymized data is available upon request at nikolaj.swiridoff@gmx.de.

## References

[1] Rönnow C.-F., Uedo N., Toth E., Thorlacius H. (2018). Endoscopic submucosal dissection of 301 large colorectal neoplasias: Outcome and learning curve from a specialized center in Europe. Endosc. Int. Open.

[2] Steinbrück I., Faiss S., Dumoulin F.L., Oyama T., Pohl J., von Hahn T., Schmidt A., Allgaier H.P. (2023). Learning curve of endoscopic submucosal dissection (ESD) with prevalence-based indication in unsupervised Western settings: A retrospective multicenter analysis. Surg. Endosc..

[3] Tutticci N., Bourke M.J. (2014). Advanced endoscopic resection in the colon: Recent innovations, current limitations and future directions. Expert Rev. Gastroenterol. Hepatol..

[4] Rahmi G., Hotayt B., Chaussade S., Lepilliez V., Giovannini M., Coumaros D., Charachon A., Cholet F., Laquière A., Samaha E. (2014). Endoscopic submucosal dissection for superficial rectal tumors: Prospective evaluation in France. Endoscopy.

[5] Repici A., Hassan C., Pagano N., Rando G., Romeo F., Spaggiari P., Roncalli M., Ferrara E., Malesci A. (2013). High efficacy of endoscopic submucosal dissection for rectal laterally spreading tumors larger than 3 cm. Gastrointest. Endosc..

[6] Probst A., Golger D., Anthuber M., Märkl B., Messmann H. (2012). Endoscopic submucosal dissection in large sessile lesions of the rectosigmoid: Learning curve in a European center. Endoscopy.

[7] Barret M., Lepilliez V., Coumaros D., Chaussade S., Leblanc S., Ponchon T., Fumex F., Chabrun E., Bauret P., Cellier C. (2017). The expansion of endoscopic submucosal dissection in France: A prospective nationwide survey. United Eur. Gastroenterol. J..

[8] Sauer M., Hildenbrand R., Oyama T., Sido B., Yahagi N., Dumoulin F. (2016). Endoscopic submucosal dissection for flat or sessile colorectal neoplasia > 20 mm: A European single-center series of 182 cases. Endosc. Int. Open.

[9] Fleischmann C., Probst A., Ebigbo A., Faiss S., Schumacher B., Allgaier H.P., Dumoulin F.L., Steinbrueck I., Anzinger M., Marienhagen J. (2021). Endoscopic Submucosal Dissection in Europe: Results of 1000 Neoplastic Lesions from the German Endoscopic Submucosal Dissection Registry. Gastroenterology.

[10] Toyonaga T., Man-i M., East J.E., Nishino E., Ono W., Hirooka T., Ueda C., Iwata Y., Sugiyama T., Dozaiku T. (2013). 1,635 Endoscopic submucosal dissection cases in the esophagus, stomach, and colorectum: Complication rates and long-term outcomes. Surg. Endosc..

[11] Saito Y., Uraoka T., Yamaguchi Y., Hotta K., Sakamoto N., Ikematsu H., Fukuzawa M., Kobayashi N., Nasu J., Michida T. (2010). A prospective, multicenter study of 1111 colorectal endoscopic submucosal dissections (with video). Gastrointest. Endosc.

[12] Spychalski M., Skulimowski A., Nishimura M., Dziki A. (2019). Comparison of Endoscopic Submucosal Dissection for Primary and Recurrent Colorectal Lesions: A Single-Center European Study. J. Laparoendosc. Adv. Surg. Tech..

[13] Steinbrück I., Faiss S., Dumoulin F.L., Oyama T., Pohl J., von Hahn T., Schmidt A., Allgaier H.P. (2023). Predictive Factors for the Outcome of Unsupervised Endoscopic Submucosal Dissection During the Initial Learning Curve with Prevalence-Based Indication. Dig. Dis. Sci..

[14] Hotta K., Oyama T., Shinohara T., Miyata Y., Takahashi A., Kitamura Y., Tomori A. (2010). Learning curve for endoscopic submucosal dissection of large colorectal tumors. Dig. Endosc..

[15] Agapov M., Dvoinikova E. (2014). Factors predicting clinical outcomes of endoscopic submucosal dissection in the rectum and sigmoid colon during the learning curve. Endosc. Int. Open.

